# Sulfophenyl-Functionalized Reduced Graphene Oxide Networks on Electrospun 3D Scaffold for Ultrasensitive NO_2_ Gas Sensor

**DOI:** 10.3390/s17122954

**Published:** 2017-12-19

**Authors:** Bin Zou, Yunlong Guo, Nannan Shen, Anshan Xiao, Mingjun Li, Liang Zhu, Pengbo Wan, Xiaoming Sun

**Affiliations:** 1 State Key Laboratory of Safety and Control for Chemicals, SINOPEC Research Institute of Safety Engineering, Qingdao 266101, China; zoub.qday@sinopec.com (B.Z.); yunlong46@126.com (Y.G.); xiaoas.qday@sinopec.com (A.X.); limj.qday@sinopec.com (M.L.); 2 State Key Laboratory of Chemical Resource Engineering, P.O. Box 98, Beijing University of Chemical Technology, Beijing 100029, China; shen_frank@126.com (N.S.); pbwan@mail.buct.edu.cn (P.W.)

**Keywords:** ultrasensitive NO_2_ sensing, 3D scaffolds, gas sensors, electrospinning, graphene

## Abstract

Ultrasensitive room temperature real-time NO_2_ sensors are highly desirable due to potential threats on environmental security and personal respiratory. Traditional NO_2_ gas sensors with highly operated temperatures (200–600 °C) and limited reversibility are mainly constructed from semiconducting oxide-deposited ceramic tubes or inter-finger probes. Herein, we report the functionalized graphene network film sensors assembled on an electrospun three-dimensional (3D) nanonetwork skeleton for ultrasensitive NO_2_ sensing. The functional 3D scaffold was prepared by electrospinning interconnected polyacrylonitrile (PAN) nanofibers onto a nylon window screen to provide a 3D nanonetwork skeleton. Then, the sulfophenyl-functionalized reduced graphene oxide (SFRGO) was assembled on the electrospun 3D nanonetwork skeleton to form SFRGO network films. The assembled functionalized graphene network film sensors exhibit excellent NO_2_ sensing performance (10 ppb to 20 ppm) at room temperature, reliable reversibility, good selectivity, and better sensing cycle stability. These improvements can be ascribed to the functionalization of graphene with electron-withdrawing sulfophenyl groups, the high surface-to-volume ratio, and the effective sensing channels from SFRGO wrapping onto the interconnected 3D scaffold. The SFRGO network-sensing film has the advantages of simple preparation, low cost, good processability, and ultrasensitive NO_2_ sensing, all advantages that can be utilized for potential integration into smart windows and wearable electronic devices for real-time household gas sensors.

## 1. Introduction

The ultrasensitive real-time detection of gaseous chemical analytes is critically important for human safety protection and environmental monitoring [1,2,3,4]. Among the various hazardous gases, NO_2_, which is mainly emitted from the fossil fuel combustion, is extremely noxious to human beings, and can lead to the photochemical smog and acid rain that threaten both environmental security and personal respiratory tracts [5,6,7,8,9]. Even the exposure of NO_2_ that is no more than 1 ppm may irritate the eyes, throat, and nose, and cause headaches or acute pulmonary edema. A threshold NO_2_ exposure of less than 200 ppb is recommended by the American Conference of Governmental Industrial Hygienists [10]. Meanwhile, traditional NO_2_ gas sensors require highly operating temperatures (200–600 °C), have limited reversibility, and are mainly constructed from semiconducting oxide-deposited ceramic tubes or inter-finger probes [11]. These result in high energy consumption and safety issues. Therefore, real-time NO_2_ sensing at room temperature is highly desirable.

Graphene materials have been widely studied in the fabrication of sensors due to their two-dimensional honeycomb structures, large theoretical specific surface areas, and high electrical conductivity [12,13]. Recently, studies have explored graphene and chemically modified graphene-coated interdigitated electrodes for NO_2_ sensing at room temperature due to their unique two-dimensional conjugated structures, excellent conductivity, and high specific surface area [14,15,16,17]. Chemically modified graphene films have good sensing performances to different concentrations of NO_2_, from 15 to 115 ppm [18]. The nitrogen-doped graphene nanosheet-based sensors demonstrated a high sensing response to NO_2_, from 2.5 to 100 ppm [19]. However, there are seldom reports for ultrasensitive sensors that detect lower concentrations of NO_2_ (10–200 ppb). Meanwhile, it has recently been well established that conductive film-based gas sensors can be potentially integrated into wearable electronics for real-time sensing [20,21]. However, chemically modified graphene-based film sensors with ultrasensitive sensing performance, room temperature chemical stability, effective portability, and excellent reversibility remain to be addressed.

**Scheme 1:**
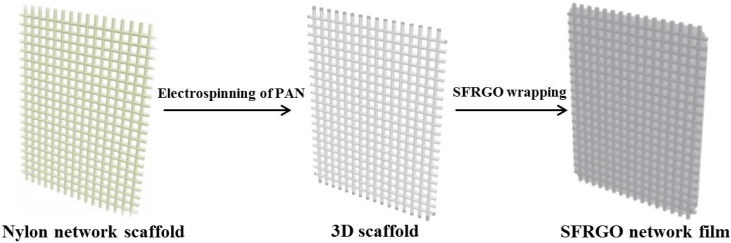
Schematic illustration for the fabrication of the sulfophenyl-functionalized reduced graphene oxide (SFRGO) network films by assembling the SFRGO onto an electrospun 3D nanonetwork skeleton.

Herein, we demonstrate the fabrication of functionalized graphene network film sensors assembled on an electrospun 3D scaffold for ultrasensitive NO_2_ sensing (Scheme 1). The functional 3D scaffold was prepared by electrospinning interconnected polyacrylonitrile (PAN) nanofibers onto a nylon window screen to provide a 3D nanonetwork skeleton. By supramolecular interaction between sulfophenyl-functionalized reduced graphene oxide (SFRGO) and PAN nanofibers, the SFRGO were assembled on the electrospun 3D nanonetwork skeleton to form SFRGO network films. The SFRGO network films were assembled as NO_2_ gas sensors. They exhibit excellent sensing performance to NO_2_ at room temperature from 10 ppb to 20 ppm, excellent reversibility, good selectivity, and better sensing cycle stability.

## 2. Materials and Methods

### 2.1. Materials

Sulfanilic acid was purchased from TCI (Shanghai) Development Co., Ltd., (Shanghai, China). Natural graphite flakes were purchased from Sigma-Aldrich (St. Louis, MO, USA). Sodium carbonate, concentrated hydrochloric acid, sodium borohydride, hydrazine (80%, N_2_H_4_·H_2_O), and sodium nitrite were purchased from Beijing Chemical Works. Polyacrylonitrile (PAN, average Mw 150,000) was purchased from HuBei ChuShengWei Chemistry Co., Ltd., (Wuhan, China).

### 2.2. Preparation of 3D Scaffold

1 g PAN was dissolved in 10 mL organic solvent (7.5 mL N,N-dimethylformamide + 2.5 mL acetone), and the mixture was stirred for 6–8 h to form a homogeneous solution. Then, the polymer solution was loaded into a 10 mL syringe, which was attached to a syringe pump (LSP01-2A, Longerpump). A nylon window screen film (15 cm × 15 cm, oxygen plasma pretreatment) coated on the aluminum foil was employed as the collector, which was placed 15 cm away from the syringe needle. The feeding rate of the solution was 0.5 mL/h, and the applied potential between the needle and collector was 20 KV [14,19]. After electrostatic spinning for different time periods, the PAN-coated nylon window screens were collected as 3D scaffolds and dried at room temperature.

### 2.3. Preparation of SFRGO

Graphene oxide (GO) was prepared from natural graphite flakes via a modified Hummer method [22]. Sulfophenyl-functionalized reduced graphene oxide (SFRGO) was synthesized from GO through an aryl diazonium reaction of sulfanilic acid [15]. 75 mg GO was added to 75 mL deionized water following ultrasonication to get a GO dispersion. Then, 5 wt% sodium carbonate solution was used to adjust the pH value to 9–10. 15 mL of 40 mg/mL sodium borohydride solution was prepared, and added into the GO dispersion at 80 °C with stirring. One hour later, the solution was centrifuged and rinsed with deionized water three to five times. Then, the obtained product was dispersed in 75 mL deionized water under sonication for 0.5 h to form a partially reduced graphene oxide (rGO) dispersion. Afterwards, 92 mg sulfanilic acid and 36 mg sodium nitrite were added into 20 mL 0.05 M HCl solution at ice-cooled conditions to get the aryl diazonium salt. Subsequently, the aryl diazonium salt was added into the partially reduced rGO dispersion and kept in an ice-water bath for 2 h. Then, the solution was centrifuged and rinsed with deionized water 3–5 times and re-dispersed into 75 mL deionized water to obtain the SFRGO solution. Finally, 25 μL hydrazine was added into the above solution, and the reaction mixture was kept at 100 °C with stirring for 24 h. The SFRGO was obtained by centrifuging and rinsing the above solution with deionized water 3–5 times and re-dispersing it into 75 mL deionized water with sonication [15].

### 2.4. Preparation of SFRGO Network Films on the Electrospun 3D Scaffold

The electrospun 3D scaffold with a size of 5 cm × 5 cm was pretreated by O_2_ plasma before effective SFRGO nanoflakes coating. Then, it was dropped into the SFRGO solution for 10 s. The process was repeated three times. After SFRGO coating, the SFRGO network films on the electrospun 3D scaffold were dried in a vacuum drying oven at 30 °C for 1 h.

### 2.5. Gas Sensing Measurement

The sensing procedure was conducted according to a previous report on the WS-30A measuring system (Zhengzhou Winsen Electronics Technology, Zhengzhou, China) for demonstrating the sensing response [20]. The functional 8 mm × 8 mm films were attached onto the probes of the voltage-testing devices with silver paint. The NO_2_ concentrations varied from 10 ppb to 20 ppm in the chamber, which could be calculated from the injected amount of NO_2_ [14]. The operating humidity was 40%, which was controlled by a SF-460 humidity controller. The sensor response (R) was defined as the normalized resistance change, R = (R_g_ − R_0_)/R_0_ = ΔR/R_0_ (R_g_: the resistance of the film after exposure to gas analytes, R_0_: the resistance of the film in the air).

### 2.6. General Techniques

SEM images were obtained using a Zeiss Supra 55 instrument at a voltage of 20 kV. Energy dispersive X-ray spectroscopy (EDX) was observed from the scanning electron microscopy with an energy dispersive X-ray. The TEM images were collected with JEOL 2100 equipment. XPS spectra were carried out by using an ESCALAB 250 model. The wavelength of the incident photon for XPS measurements was 1253.6 eV, and the source of the X-rays for XPS measurements was Ma Kα. Raman spectra were collected from a JobinYvon Lab RAM HR Raman microscope with an excitation wavelength of 532 nm.

## 3. Results

### 3.1. The Morphology and Structure

**Figure 1:**
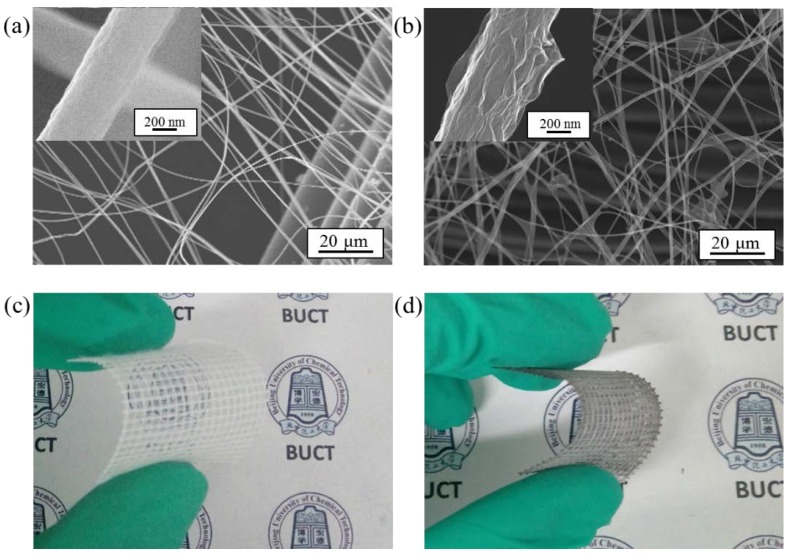
SEM images of (**a**) the electrospun polyacrylonitrile (PAN) nanofiber skeleton (inset: the high-resolution SEM image), (**b**) the wrapped SFRGO onto the PAN nanofiber skeleton (inset: the high-resolution SEM image). Photographs of (**c**) the electrospun PAN nanofibers on the nylon scaffold, and (**d**) the flexible SFRGO network films. The school badges are placed behind the film.

After electrospinning PAN onto nylon window screens for 0.5 h, 1.0 h, and 1.5 h, the PAN-coated nylon window screens were obtained as 3D scaffolds with different densities and dried at room temperature. As shown in Figures S1 and S2, when the electrospinning time increased, the PAN gradually became much denser. Then, the as-prepared 3D scaffolds with different electrospinning times were dipped into the SFRGO solution for SFRGO wrapping (Figure S3) onto the PAN nanofibers through supramolecular ion–dipole interactions between the polar groups (–CN) of the polymer and the ionic salt group of SFRGO (Figure 1 and Figure S4) [23]. As shown in Figure 1a, a porous interwoven structure with the randomly oriented PAN nanofibers (the diameter at 434 nm) were observed. After wrapping SFRGO onto the PAN nanofibers, the SFRGO sheets were uniformly coated onto the PAN nanofibers and the nanofibers’ junctions, resulting in continuous conducting pathways, as confirmed by the SEM image in Figure 1b. The denser SFRGO sheets were deposited onto the electrospun PAN nanofibers with more electrospinning time (Figure S4).

**Figure 2:**
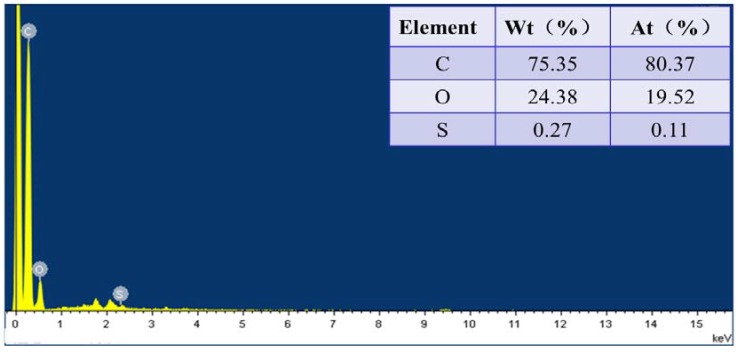
The energy-dispersive X-ray spectroscopy (EDX) spectra of sulfophenyl-functionalized reduced graphene oxide (SFRGO).

**Figure 3:**
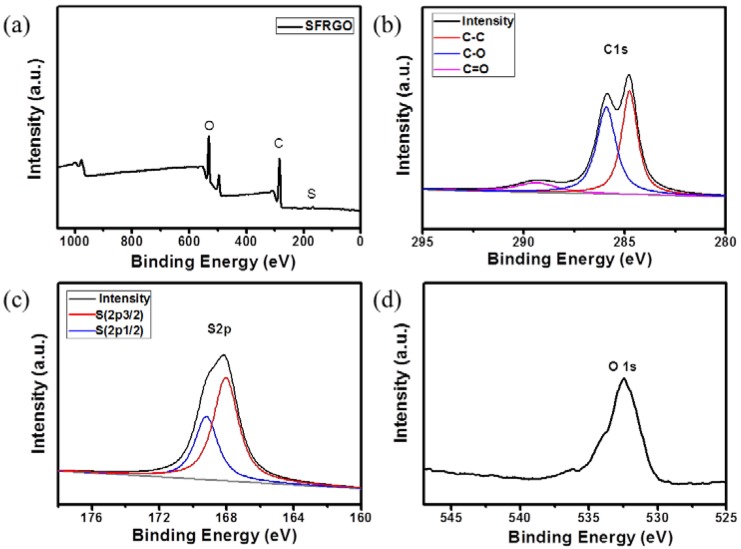
(**a**) The X-ray photoelectron spectroscopy (XPS) spectra of SFRGO. XPS spectra for (**b**) C 1s, (**c**) S2p, and (**d**) O 1s.

**Figure 4:**
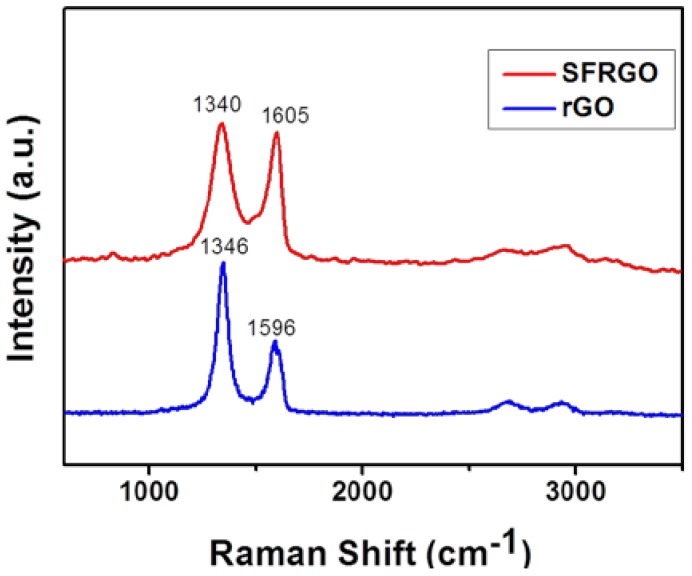
Raman spectra of SFRGO and reduced graphene oxide (rGO).

The chemical composition of the obtained SFRGO sheets was characterized by energy-dispersive X-ray spectroscopy (EDX) (Figure 2) and X-ray photoelectron spectroscopy (XPS) (Figure 3). As demonstrated in Figure 2, the elements Sulfur (S), carbon (C), and oxygen (O) were observed. As displayed in Figure 3, the peaks for C 1s and O 1s were observed at 282 eV and 529 eV, respectively, and the peak for element S was presented at 168 eV. Figure 3b exhibited the main C 1s peaks centered at 284.8, 286.7, and 288.6 eV for the C–C, C–O, and C=O species, respectively. The peaks at 161.7 eV and 168.4 eV were attributed to the –SO_3_H and the high oxidized S, respectively [14]. The peaks for the S (2p_3/2_) species and the S (2p_1/2_) species at 167.5 eV and 168.7 eV are shown in Figure 3c [24,25,26]. Thus, these results indicated the presence of the sulfophenyl group in SFRGO sheets. Figure 4 displayed the Raman spectra of SFRGO and rGO. The Raman spectrum of SFRGO showed two typical peaks at 1340 cm^−1^ (D mode due to defects) and 1605 cm^−1^ (G mode due to E_2g_ vibration). The value of D mode intensity to G mode intensity (I_D_/I_G_) was 1.10, which was lower than that for rGO (1.76) due to the limited π-conjugated structures of SFRGO from the modification of the sulfophenyl group [14,15,27,28,29].

### 3.2. Gas-Sensing Properties

**Figure 5:**
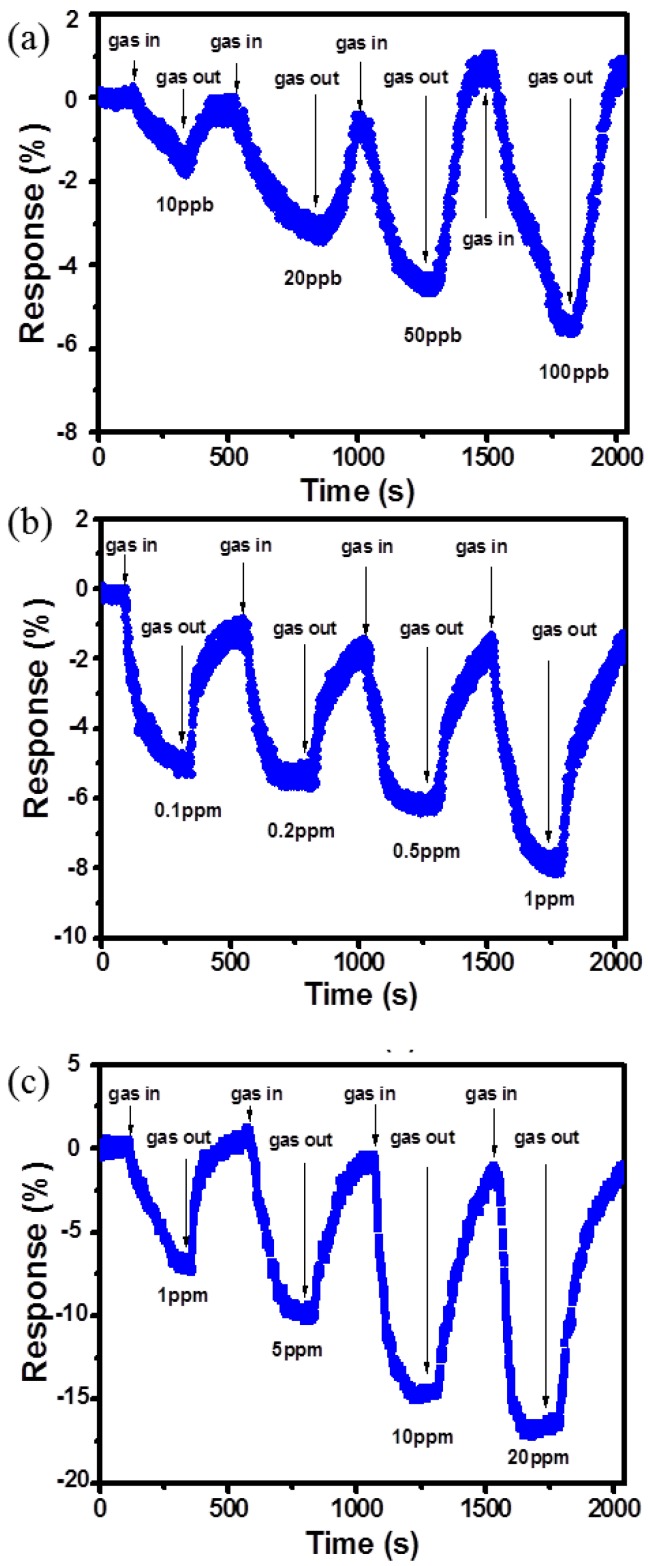
Sensing performance of the SFRGO network film devices to different concentrations of NO_2_, from 10 ppb to 20 ppm ((**a**) 10–100 ppb; (**b**) 100–1000 ppb; (**c**) 1 ppm–20 ppm).

**Figure 6:**
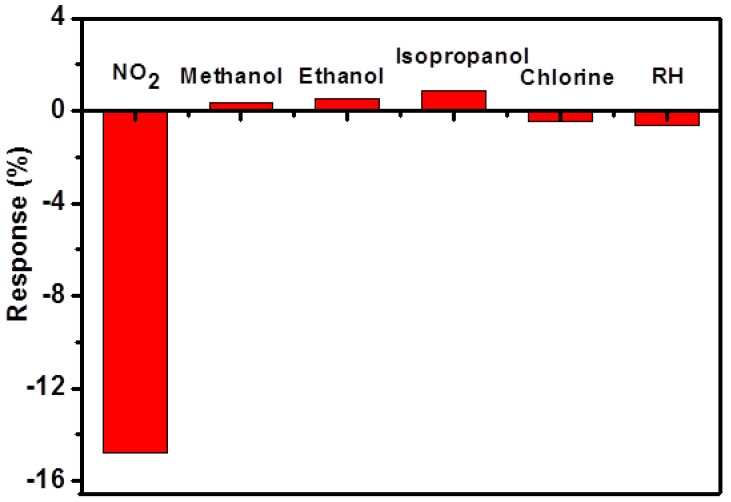
Gas sensing selectivity of the SFRGO network film devices to 10 ppm NO_2_ and 25 ppm methanol, 25 ppm ethanol, 25 ppm isopropanol, 25 ppm chlorine, and 50% relative humidity.

**Figure 7:**
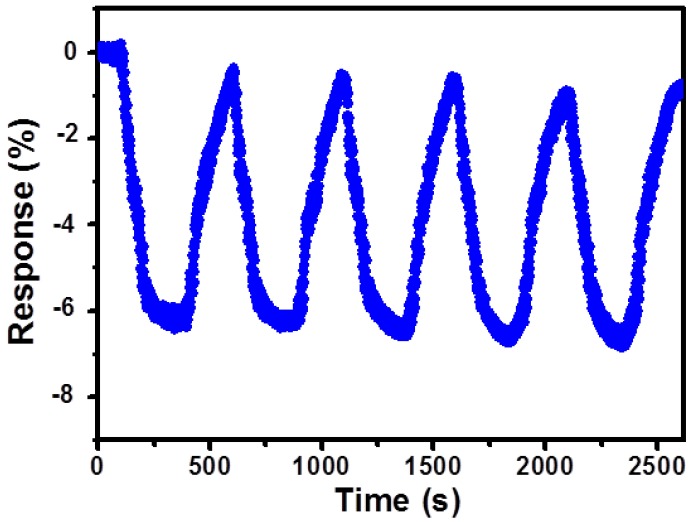
The sensing repeatability for the SFRGO network film devices to 500 ppb NO_2_.

We wondered whether the obtained SFRGO network films could be assembled as gas sensors for ultrasensitive NO_2_ at room temperature. In order to resolve this issue, the sensing performances of the obtained SFRGO network films for NO_2_ were tested (Figure 5 and Figure S5). Figure S5 displayed the sensing responses of SFRGO networks wrapped onto the electrospun PAN nanofiber scaffolds with different electrospinning times. These responses indicated the optimal sensing performance to NO_2_ for the SFRGO networks that had been wrapped onto the electrospun PAN nanofiber scaffolds with 1 h electrostatic spinning. For the samples prepared on the electrospun PAN nanofiber scaffolds with an electrospinning time of less than 1 h, the lower sensing response was probably ascribed to the less deposited sensing material, SFRGO, onto the 3D scaffolds, the lower surface area, and the less interconnected network sensing channels. For the samples prepared on the electrospun scaffolds with an electrospinning time that was higher than 1 h, denser SFRGOs were deposited, resulting in the decreased exposed active surfaces, and invalid network sensing channel. Figure 5 demonstrates the NO_2_ sensing response for the SFRGO networks that had been wrapped onto the electrospun PAN nanofiber scaffolds (electrostatic spinning for 1 h) to different concentrations of NO_2_ from 10 ppb to 20 ppm at room temperature. As shown in Figure 5, the sensing response increased gradually with the increased NO_2_ concentration. The resistance change of the SFRGO network films could be still observed under NO_2_ concentrations as low as 10 ppb (Figure 5a), indicating its superior trace NO_2_ sensing in chemical detection. Figure S6 displays the comparison of the sensing responses between rGO and SFRGO, and indicated that the SFRGO network film has a better sensing response to NO_2_. The sheet resistances of the rGO and SFRGO films were 1831 kΩ/□ and 1310 kΩ/□, respectively. Meanwhile, the sensing performances upon exposure to different gases, including methanol, ethanol, isopropanol, and chlorine are shown in Figure 6, and also exhibited a relatively low response to 50% relative humidity (RH). These results also implied the excellent sensing selectivity to NO_2_ for the SFRGO network films. The sensing repeatability of the SFRGO network film device was tested for sensing 500 ppb NO_2_ under five successive cycles in Figure 7, and these results also demonstrated its excellent sensing stability and reversibility to NO_2_ sensing.

### 3.3. Sensing Mechanisms to NO_2_

The superior sensing performances of the SFRGO network film devices could be attributed to the effective chemical modification of rGO with the electron-withdrawing group and the efficient wrapping onto the 3D nanonetwork skeleton, which provided continuous sensing pathways. After the functionalization of p-type rGO [30] with electron-withdrawing sulfophenyl groups, the SFRGO sheets became more hole-doped. The electron-rich sites—for example, the lone-pair electrons of S or O atoms in sulfophenyl groups—tend to adsorb the electron-withdrawing molecule NO_2_. Thus, upon NO_2_ adsorption, the conductance of the SFRGO increased greatly due to the better p-type doping level of the rGO sheets from the electron-withdrawing sulfophenyl group [14,15]. Meanwhile, the high surface-to-volume ratio and the effective continuous sensing channels from the efficient SFRGO wrapping onto the interconnected 3D nanonetwork scaffold, could contribute better sensing stability and excellent reversibility to the ultrasensitive NO_2_ sensing [16,31,32,33].

## 4. Conclusions

In conclusion, we have fabricated ultrasensitive NO_2_ film sensors from the effective SFRGO wrapping onto the electrospun 3D scaffolds. The assembled film sensors exhibited an excellent NO_2_ sensing performance at room temperature from 10 ppb to 20 ppm, good reversibility, and better sensing repeatability. These improvements can be ascribed to the functionalization of rGO with electron-withdrawing sulfophenyl groups, the high surface-to-volume ratio, and the effective continuous sensing channels from the efficient SFRGO wrapping onto the interconnected 3D nanonetwork scaffold. The SFRGO network sensing film has the advantages of simple preparation, low cost, good processability, and ultrasensitive NO_2_ sensing, which may be potentially assembled onto smart windows and wearable electronic devices for real-time household gas sensors.

## Supplementary Materials

The following are available online at http://www.mdpi.com/1424-8220/17/12/2954/s1.
