# Supplementary material for: Sulfophenyl-Functionalized Reduced Graphene Oxide Networks on Electrospun 3D Scaffold for Ultrasensitive NO2 Gas Sensor
Source: Sensors (Basel). 2017 Dec 19;17(12):2954. doi: 10.3390/s17122954 (PMC5751658; doi:10.3390/s17122954)
Supplement: Supplementary file 1 [file sensors-17-02954-s001.pdf]

Electronic Supplementary Material (ESI)

*Article*

# Functionalized Graphene Networks on Electrospun 3D Scaffold for Ultrasensitive $NO_2$ Gas Sensor

**Bin Zou [1], Yunlong Guo [1,2], Nannan Shen [2], Anshan Xiao [1], Mingjun Li [1], Liang Zhu [1,*], Pengbo Wan [2], Xiaoming Sun [2,*]**

[1] State Key Laboratory of Safety and Control for Chemicals, SINOPEC Research Institute of Safety Engineering, Shandong Qingdao 266101, P.R. China; zoub.qday@sinopec.com (B. Z.); yunlong46@126.com (Y. G.); xiaoas.qday@sinopec.com (A. X.); limj.qday@sinopec.com (M. L.); zhul.qday@sinopec.com (L. Z.)

[2] State Key Laboratory of Chemical Resource Engineering, P.O. Box 98, Beijing University of Chemical Technology, Beijing 100029, P.R. China; yunlong46@126.com (Y. G.); shen_frank@126.com (N. S.); pbwan@mail.buct.edu.cn (P. W.); sunxm@mail.buct.edu.cn (X. S.)

* Correspondence: zhul.qday@sinopec.com (L. Z.); sunxm@mail.buct.edu.cn (X. S.); Tel.:+86-10-64448751 (X. S.)

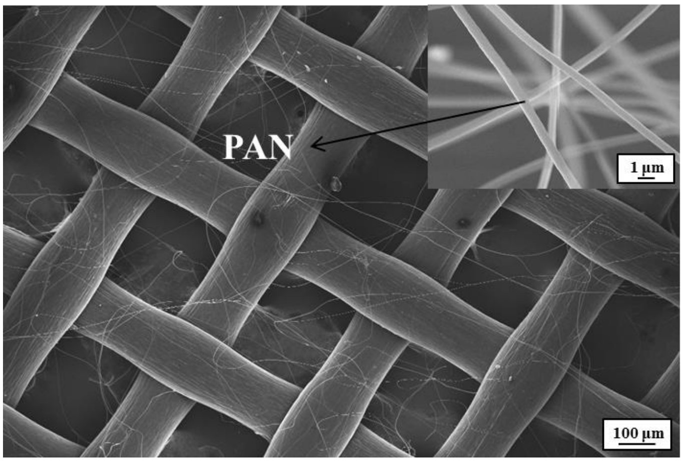


**Figure S1.** SEM image of electrospun PAN onto nylon window screens for 0.5 h.

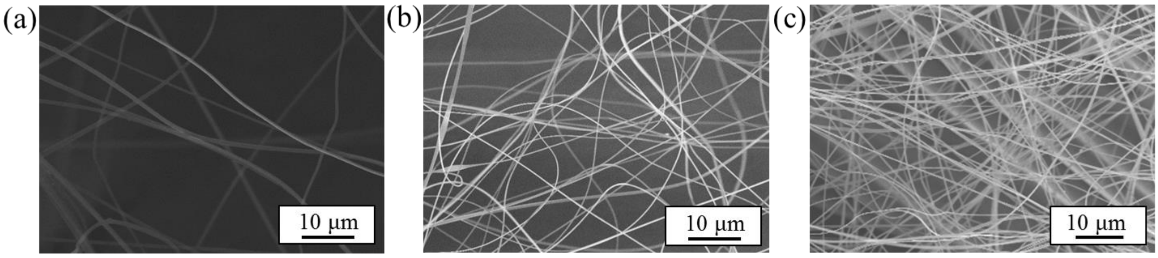


**Figure S2.** SEM images of electrospun PAN onto nylon window screens for (a) 0.5 h, (b) 1.0 h, and (c) 1.5 h, respectively.

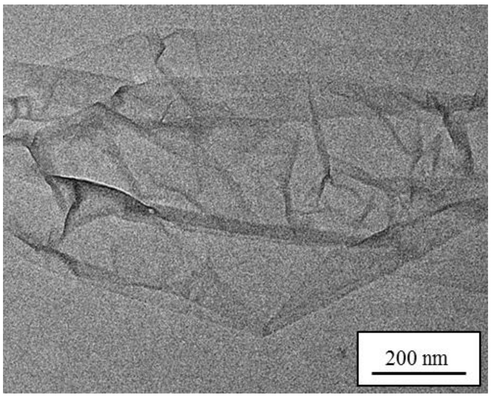


**Figure S3.** TEM image of SFRGO.

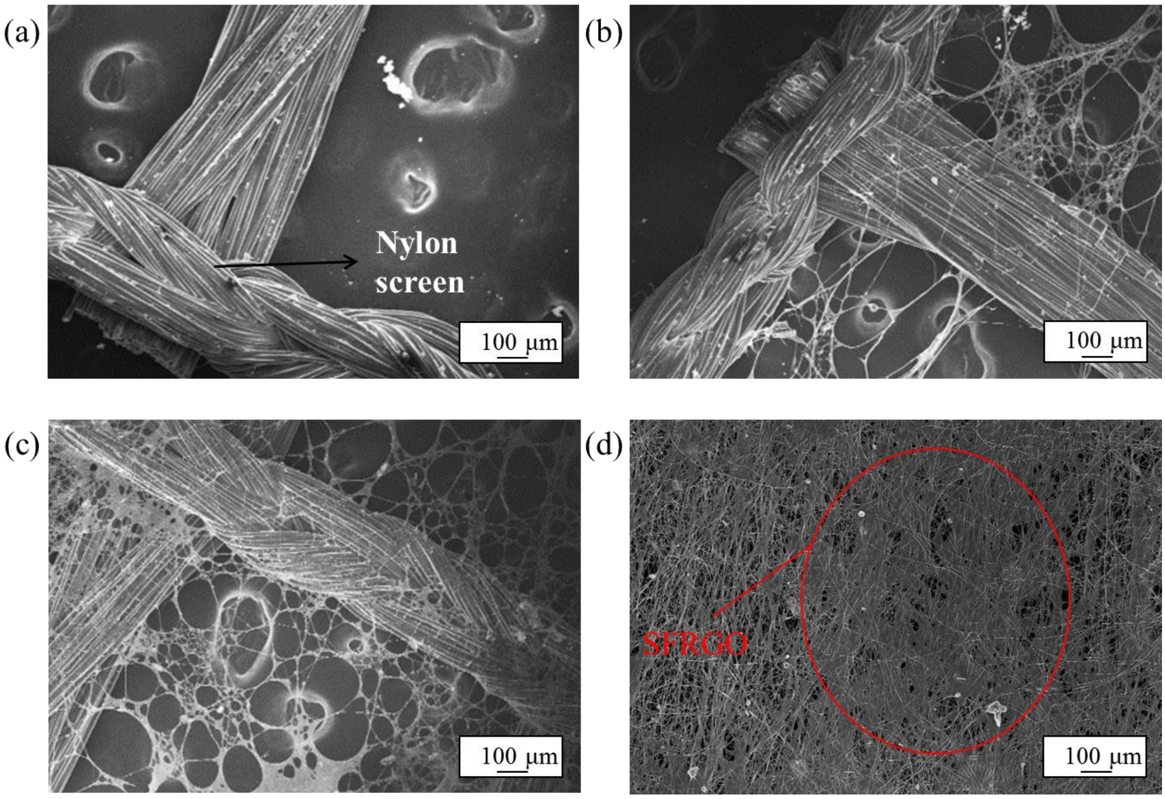


**Figure S4.** SEM images of SFRGO wrapping onto the electrospun PAN nanofibers with elelctrospinning time at (a) 0 h, (b) 0.5 h, (c) 1.0 h, (d) 1.5 h.

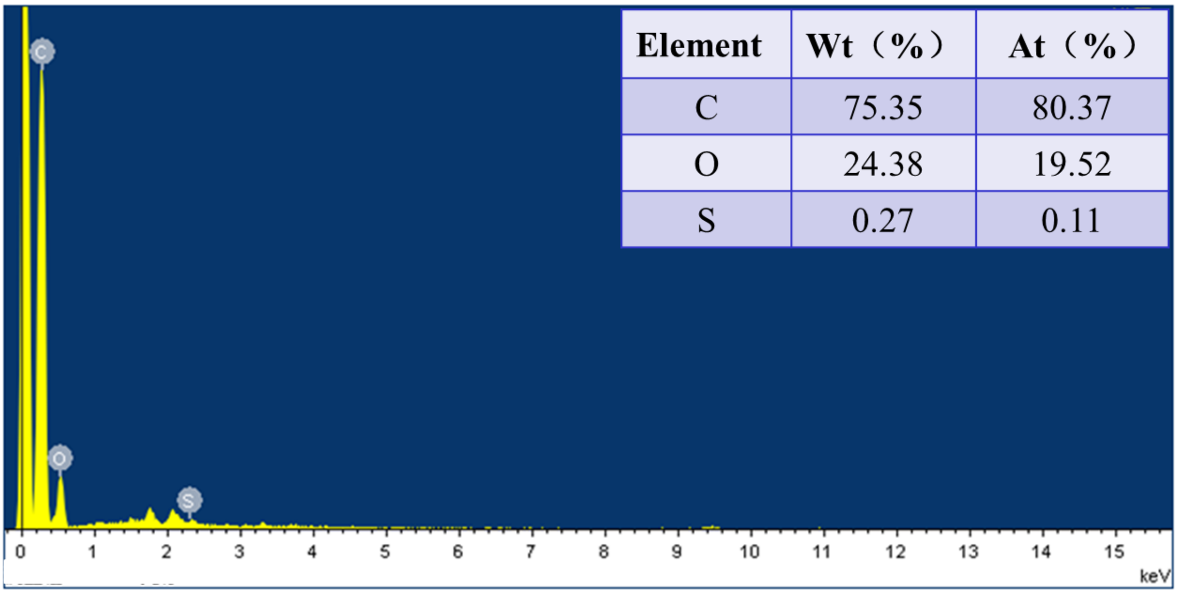


| Element | Wt（%） | At（%） |
|---|---|---|
| C | 75.35 | 80.37 |
| O | 24.38 | 19.52 |
| S | 0.27 | 0.11 |

**Figure S5.** The EDX spectra of SFRGO.

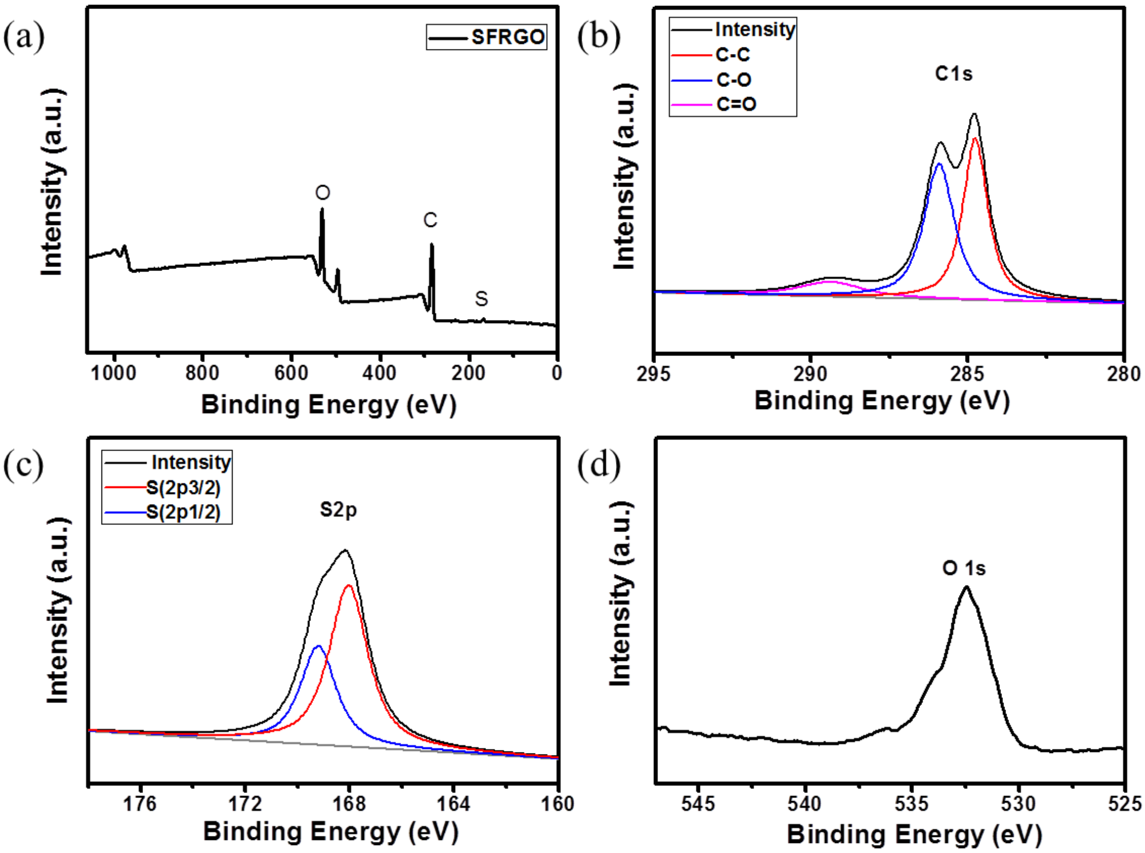


**Figure S6.** The XPS spectra of SFRGO.

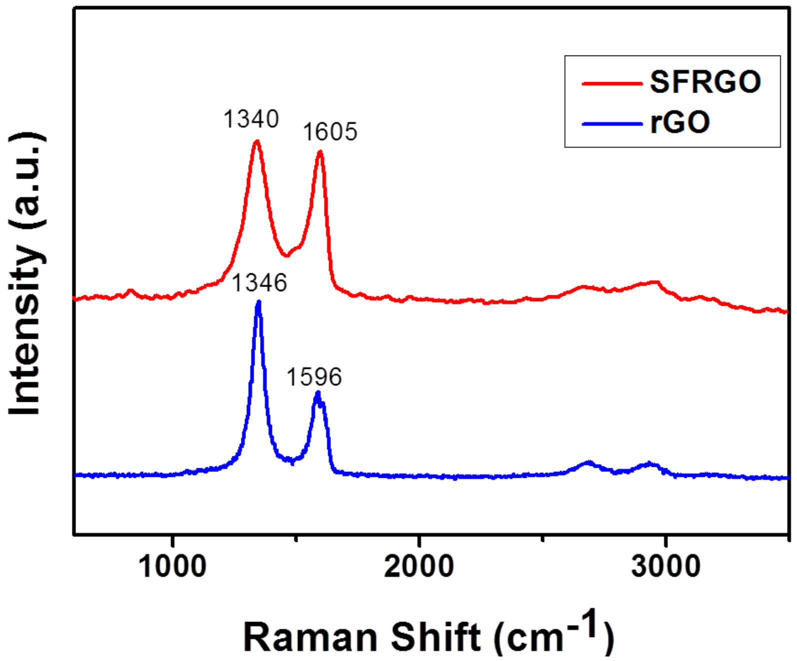


**Figure S7.** Raman spectra of SFRGO and rGO.

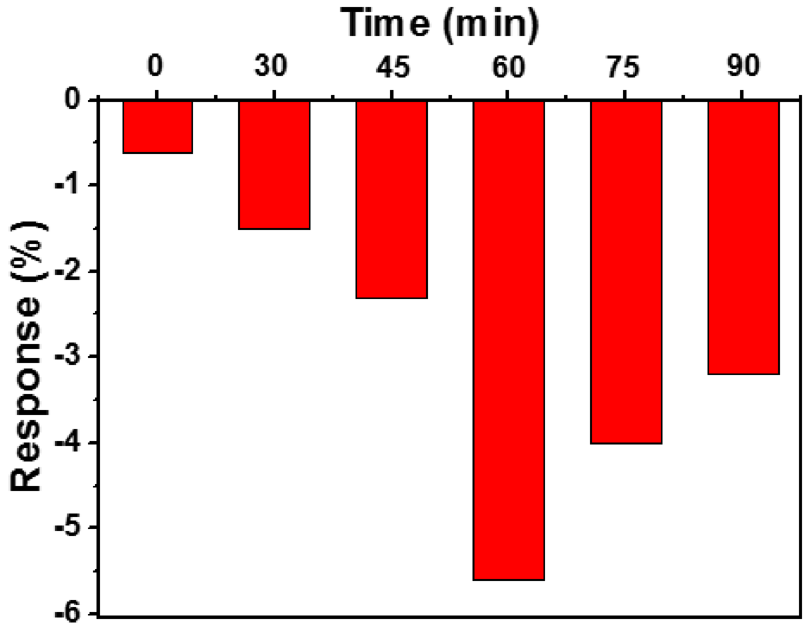


**Figure S8.** Sensing responses of SFRGO networks wrapped onto the electrospun PAN nanofiber scaffolds with different elelctrospinning time.
